# Improvement of Psoriasis Using Oral Probiotic *Streptococcus salivarius K-12*: a Case–Control 24-Month Longitudinal Study

**DOI:** 10.1007/s12602-022-09937-1

**Published:** 2022-04-13

**Authors:** Arianna Zangrilli, Laura Diluvio, Arianna Di Stadio, Stefano Di Girolamo

**Affiliations:** 1 Dermatology, Department of Systems Medicine, University of Rome Tor Vergata, Viale Oxford, 8100133 Rome, Italy; 2 Department of Otorhinolaryngology, University of Rome Tor Vergata, Viale Oxford, 8100133 Rome, Italy; 3 Department G.F Ingrassia, University of Catania, Catania, Italy

**Keywords:** Psoriasis, *Streptococcus salivarius*, Oral probiotic, Treatment, Outcome, Long-term efficacy

## Abstract

Psoriasis is a common chronic skin disease, associated with an important physical and physiological involvement for any age. There is a strong link between psoriasis and streptococcal infection, particularly that of the tonsils. There are many therapies to treat psoriasis including topical, systemic, and biologic agents but these treatments are not free from side effects. *Streptococcus salivarius K-12* is an oral probiotic product useful for the prophylaxis and treatment of tonsillar infections in children and adults, now tested here for the first time for control of psoriasis. Our retrospective analysis was conducted on 198 patients affected by mild to moderate psoriasis: 100 patients were first treated for 90 days with *Streptococcus salivarius K-12*, while 98 did not receive any probiotics and were the control group. The patients treated with *S. salivarius K-12* exhibited a significant improvement of their psoriasis from the baseline condition: 83.7% patients treated achieved a 100% improvement of the PASI score at 24 weeks and efficacy continued to improve with longer treatment, maintaining same result also during follow-up observation. In all patients, the treatment was well tolerated, and no adverse events have been observed. Our data show that oral preparations containing *Streptococcus salivarius* may provide a beneficial option for the prevention and cure of pediatric and adult psoriasis.

## Introduction

Psoriasis is a common, chronic skin disease, affecting approximately 2% of the population and is mainly a dendritic cell and T-cell-mediated disease with complex feedback loops from antigen presenting cells, neutrophilic granulocytes, keratinocytes, vascular endothelial cells, and the cutaneous nervous system. Crosstalk between the innate and the adaptive immune system mediated by cytokines including TNF-α, interferon-γ, and interleukin-1 is a major research focus [1, 2]. The dermal plasmacytoid dendric cell seems to be sensitive to the stimulation of complexes of host DNA and the epidermis-produced antimicrobial peptide LL-37, which induce these cells to produce interferon-α [3]. Activated dendritic cells produce TNFα and interleukin both on the onset and the relapsing of psoriasis [4]. Five types of psoriasis have been reported: plaque psoriasis (also known as psoriasis vulgaris) [5]; guttate (droplet) or eruptive psoriasis, characterized by scaly teardrop-shaped spots; inverse psoriasis (intertriginous or flexural psoriasis) usually found in folds of skin; pustular psoriasis, which can be as palmoplantar pustulosis (pustular psoriasis of the palms and soles), or generalized pustular psoriasis (a rare and serious form of psoriasis); and erythrodermic psoriasis, which is a rare but very serious complication of psoriasis [6–12]. *Streptoccoccus pyogenes* frequently causes recurrent pharyngo-tonsillar infections in young children, and this is associated with the further requirements for recurrent clinical examinations, pharmacological treatments, specialist consultations, and sometimes surgical intervention [13, 14]. Many patients with psoriasis suffer from relapsing of the symptoms when affected by upper respiratory infection [13]. It has been hypothesized that persistence of streptococcus may be the trigger (molecular mimicry) for the activation of skin-homing T cells. In fact, the researchers observed that tonsils from patients with psoriasis contain more T cells with skin homing potential compared with non-psoriatic individuals. Furthermore, superantigens produced by β*-hemolytic streptococci* can enhance T-cell expression of cutaneous lymphocyte-associated antigen (CLA)–a carbohydrate moiety expressed on 80% of T cells in the skin–and the frequency of CLA + CD8 + T cells in patients; these two conditions can correlate with patients disease severity. Although the relevance of specific effector/memory T cells is well known, it is still unclear how and why the effector/memory T cells are activated. The innate immune response of the tonsils seems to play a crucial role in stimulating and regulating the T-cell-mediated adaptive immune response. An excessive/aberrant tonsillar response to the streptococcal infection could cause T-cell expansion followed by movement of these cells to the skin. Once here, T-cells could induce an inflammatory response responsible for the relapsing/onset of psoriasis plaque. This finding is supported by the presence of oligoclonal T cells both in the tonsils and psoriatic plaques of the same individual, with disease remission after tonsillectomy [15]. The research investigating the inflammation in the upper respiratory tract identified a close link between the lowering of potential bacterial pathogens and the presence of “antagonistic” streptococcal commensals in the microbiota; it has been shown that probiotic organisms can confer the same level of protection as naturally harbored strains [16]. *Streptococcus salivarius K-12* is an oral probiotic, recently available in Italy for pediatric and adult use. *S. salivarius K-12*, a predominant colonizer of the oral cavity, produces two bacteriocins, salivaricin A2 and salivaricin B, both of which antagonize the growth of *Streptoccoccus pyogenes*.

*S. salivarius* can also inhibit growth of *Haemophilus influenzae*, *Streptococcus pneumoniae*, *Moraxella catarrhalis*, *Micrococcus luteus*, *Streptococcus anginosus*, *Eubacterium saburreum*, and *Micromonas micros* [16]. Strain of *S. salivarious* are used as probiotic and produces multiple bacteriocins and it used to reduce levels of *Streptococcus salivarius*, *Streptococcus mutans*, *lactobacilli*, *β-haemolytic streptococci*, and *Candida* species. Bacteriocins represent antimicrobials with specific killing activity [17]. They suppress the growth of bacteria that are phylogenetically closely related to the bacteriocin-producing strain. Unlike antibiotics, the spectrum of action of bactericins does not include microbial species that are distanced phylogenetically from the producer strain [16]. The aim of the present study is to investigate the efficacy and safety of oral probiotic treatment with *Streptococcus salivarius K12* (Bactoblis ®; Pharmaextracta SpA, Pontenure (PC), Italy) after restoration of the natural conditions of the microbiota and the achievement of the persistence of probiotics in the upper respiratory tract on the clinical manifestations of psoriasis in children and adults who had a recent history of recurrent episodes of pharyngo-tonsillar infections [18].

## Materials and Methods

This prospective study aimed to evaluate the efficacy and safety profile of orally administered probiotic *Streptococcus salivarius K-12* in patients affected by mild/moderate psoriasis. The data were collected in a tertiary University Hospital (Tor Vergata) collection over 10-month period, between October 2017 and August 2018. All patients signed a written consent before being included and authorized the use of their data for scientific purpose. The study was approved by the hospital Review Board on 15/09/2017 with number 092017PTV. The study was in accordance with the ethical principles of the Declaration of Helsinki and was consistent with the guidelines for good clinical practice. The study included a total of 196 patients over 7 years of age (age 7 ± 67.5 year; 92 M/100 F).

Inclusion criteria were psoriasis vulgaris (prevalent type and guttate psoriasis), history of streptococcal infections of tonsils and possibility of test antistreptolysin O-titer (ASLO titer) (normal < 200 UI/ml), and antistreptococcal antibody (normal < 85 Ui/ml) to identify recent strep infection.

Exclusion criteria were a concurrent diagnosis of other immunological disorders: Crohn’s disease, ulcerative colitis, rheumatoid arthritis, ankylosing spondylitis, subjects with onset of a severe organ dysfunction, terminal illness, human immunodeficiency virus (HIV) infection or cancer throughout the study duration, patients treated with antibiotics during the last 4 weeks.

Washout periods of 30 days or 5 half-lives–time to reduce the plasma concentration of a drug until considering it as eliminated–(which ever was longer) were required for biological, systemic, and investigational agents prior to baseline.

The subjects who met inclusion criteria were randomized and assigned to the treatment or control group. Ninety-eight patients (treatment group) were treated for 90 days with an oral supplement that contains 1 billion of *Streptococcus salivarius K12* (1 tablet daily) [19] and topical treatment such as emollient and vitamin D derivatives, while 98 subjects (control) with topical treatment such as emollient and vitamin D derivatives (Table 1). *S. salivarius* was administered as tablets sucked slowly in the evening just before bedtime, one tablet each day for 24 weeks in psoriatic patients. Every tablet contains one billion colony-forming units (CFU) *S. salivarius*/dose (based on the product expiry date).

**Table 1:** Demographic characteristics of treatment and control group

**Characteristics of the study population (*n* = 198)**	
Gender	Females 102
	Males 96
Age (years)	Age 7 ± 67.5 year
Smoking	52
Psoriasis duration (years)	2 ± 24 year
Previous phototherapy	23
Previous cyclosporin	52
Previous acitretin	45
Previous methotrexate	62
Palmoplantar	30
Guttate psoriasis	81
Scalp psoriasis	89
Genital psoriasis	12
Facial psoriasis	24

The patients were evaluated at baseline and then after twenty-four weeks by calculating the Psoriasis Activity and Severity Index (PASI) [20] and the Dermatology Life Quality Index (DLQI) [19]. Efficacy of probiotic product to reduce psoriatic lesions was evaluated at each follow-up visit as PASI 75/90/100 response rates (i.e., percentage of patients with a reduction of the baseline PASI score ≥ 75%/ ≥ 90% [clear or almost clear skin]/ = 100% [clear skin], respectively).

All endpoints were evaluated at week 0 and after 24 weeks of treatment. Adverse events were evaluated at every study visit and up to 60 days following the last dose of study medications.

All medications received at the time of screening and enrollment or during the study were recorded.

### Statistical Analysis

Data were summarized as mean and standard deviation for continuous variables and as number of patients (percentage) for each categorical variable. Chi-square (*χ*) was performed to evaluate the statistical difference between treatment and control group. The statistical significance was set at *p*-value < 0.05. The analysis was performed by PSPP ® statistical software.

## Results

**Fig. 1:**
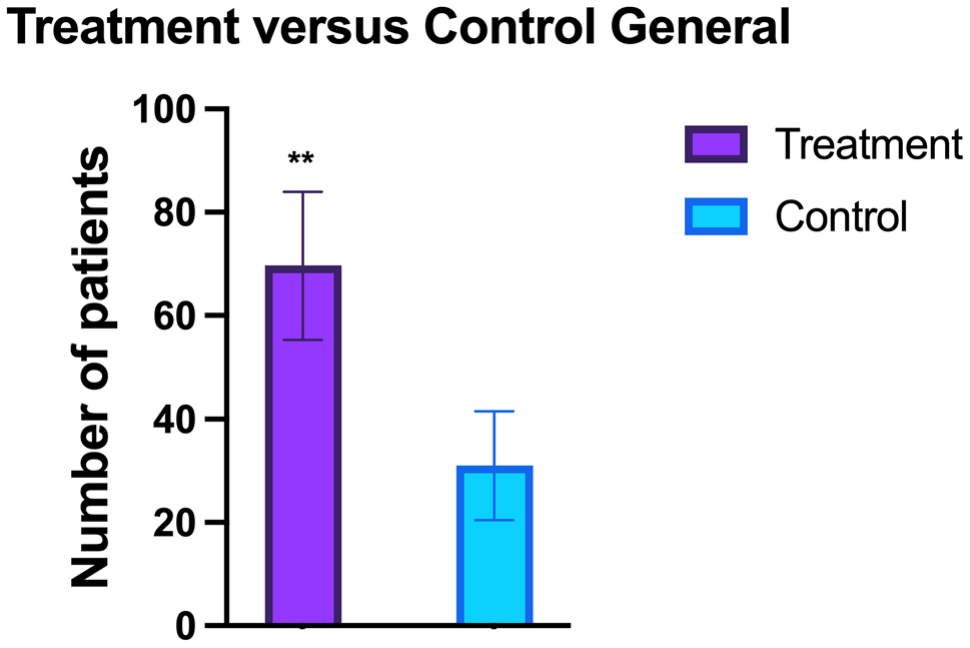
The graph shows the statistically significant differences (** = 0.01) identified between the treatment and control group in absolute. indicates standard deviation (SD)

**Fig. 2:**
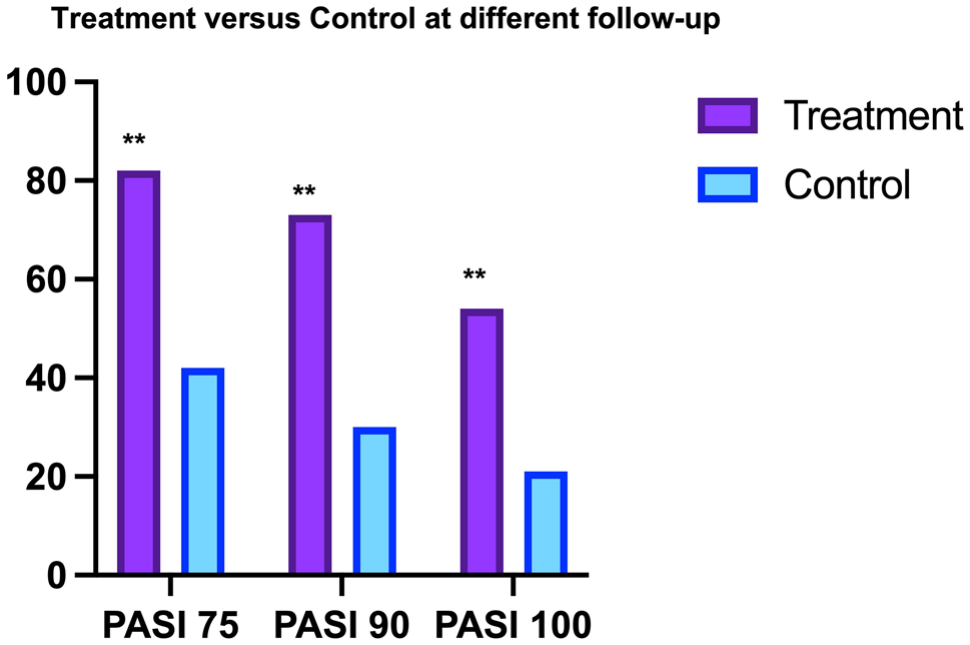
The differences between treatment and control are statistically significant at any of the observation point (** = 0.01)

**Fig. 3:**
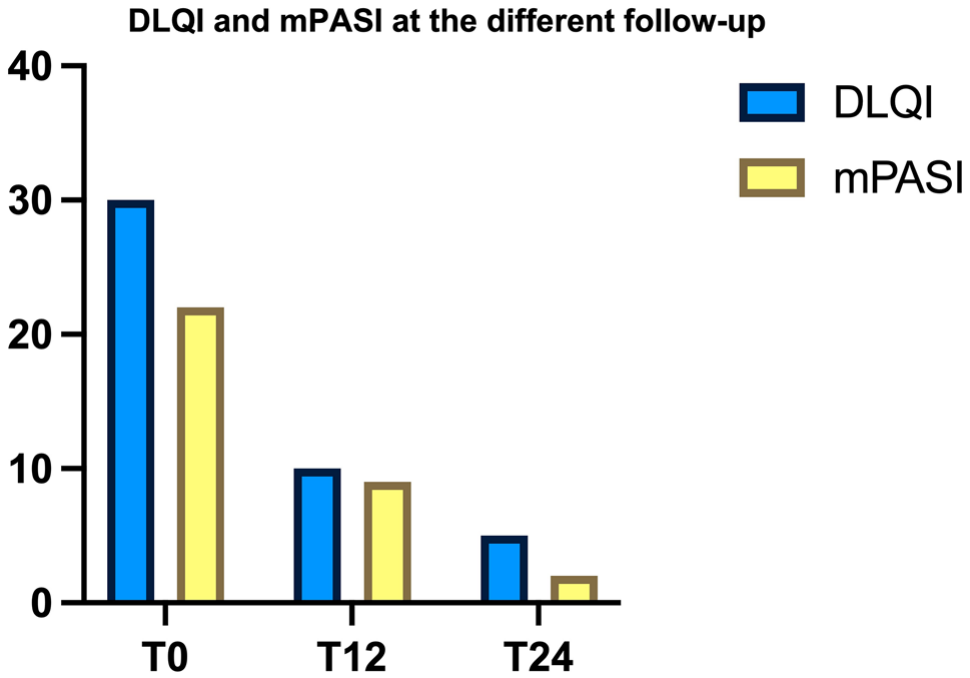
The graph reports the results of DLQI and mPASI at T0, T12, and T24

In the treatment group, mean baseline PASI score was 17.3 ± 15.4 (SD = 5683), compared to the control group was 7.5 ± 6.5 (SD = 3092). At week 24, PASI 75 response was achieved by 84% (*n* = 84) of patients, PASI 90 response was achieved by 75% of patients (*n* = 75), and PASI 100 response was achieved by 55% of patients (*n* = 55) (SD = 1364). At the same point, 42.8% of control group (*n* = 42) achieved PASI75, PASI 90 response was achieved by 30.6% of patients (*n* = 30), and PASI 100 response was achieved by 21.4% of patients (*n* = 21) (SD = 2494). Statistically significant variances were observed comparing treatment and control group (*χ*; *p* = 0.01) (Figs. 1 and 2). The patients treated with *S. salivarius K-12* efficacy continued to improve with longer treatment, maintaining same result also during follow-up observation. In all patients, the treatment was well tolerated, and none of the patients had adverse events (Fig. 3). Moreover, despite the treated patients having ASLO and antistreptococcal antibody titers positivity at the start of therapy (meaningful that the patients had had an infection in the previous weeks currently solved), they achieved PASI100 after 24 weeks the same, instead in the control group only 66.7%.

By comparison with the control group, the patients treated with *S. salivarius* experienced a significant improvement of the skin pathology and they did not have a worsening even after discontinuation of treatment throughout the follow-up period. Our analysis has also demonstrated the absence of any dependency on sex and age variables. The safety profile during the observational period did not report any side effects and no probiotic-related infections.

## Discussion

In most patients, especially individuals who carry the human leukocyte antigen (HLA)-C0602 allele, first initiation and acute exacerbation of psoriasis are triggered by tonsillar infection with *Streptococcus pyogenes* [21]. The incidence of preceding streptococcal sore throat in psoriasis ranges between 58 and 97% [6, 22]. *Lancefield group A streptococci* (*S. pyogenes*) and streptococci of groups C and G have been also found in the patients’ tonsils, and could act as trigger; in particular, the streptococci express on their surface several antigenically distinct M proteins. This protein is essential for bacterial virulence, providing antiphagocytic functions critical to survival in human tissues and fluids. Specific regions of this protein serve as shared antigens, and cross-reactivity between these epitopes and human proteins may be responsible of the autoimmune sequelae such as rheumatic heart disease [23].

The streptococci are internalized in the epithelial tonsillar cells, in which they can stay for long becoming a reservoir of potentially disease-stimulating antigens. Two years after tonsillectomy, the patients still suffer from relapse of psoriasis; it could be related to the Streptococcal colonization by *streptococci* of other tissues in the upper respiratory tract. Streptococci superantigens could induce skin-seeking T cells in lymph nodes, and these cells move in the skin where they might ulteriorly proliferate. Some patients suffer from frequent psoriasis relapse when affected by tonsillitis, so the antigen priming of T cells may occur in the skin-draining lymph nodes and/or in the tonsils. This can be confirmed by the presence of the same clonal T cell receptor rearrangements both in lesioned skin and tonsil of the same patient. A streptococcal antigen or a skin specific antigen with or without homology to a streptococcal antigen might be responsible for activation of these potentially pathogenetic T cells in the skin [24]. *Streptococcus pyogenes* might induce an autoimmune disease due immunological mimicry between molecules expressed by the bacterium and target tissue [25, 26]. Moreover, this association has been clinically confirmed by improvement of psoriatic lesions after tonsillectomy or antibiotic treatment [27]. It is well recognized the effect of prophylactic administration of *S. salivarius* to pediatric patients and adult patients having a history of recurrent oral streptococcal pathology reduced the number of episodes of streptococcal pharyngeal infections and/or tonsillitis and/or dental caries and/or cystic fibrosis and probiotic treatment with *Streptococcus salivarius* to reduce the incidence of pharyngo-tonsillitis caused by *Streptococcus pyogenes* [16, 28–31]. Currently, there are many therapies to treat psoriasis such as topical, systemic, and biologic agent but these treatments are not free from side effects. Moreover, due to the poor literature on the use of biologic agents in childhood psoriasis, safety data from controlled trials is usually only available for the most sensitive indication, for only one dose and for only one age group. This study shows that psoriasis can also be treated by preventing streptococcal infection without side effects and at any age. It is strongly recommended to support the efficacy and safety of probiotics in psoriasis with clinical data. A large number of patients cannot be treated with systemic psoriasis medications due to contraindications or side effects; they would benefit from treatment with the probiotics. This oral probiotic treatment will allow better management of psoriasis diseases to improve the quality of life of the patient. The patients treated with oral probiotics *S. salivarius* had a significantly improvement of psoriasis from baseline and they did not have relapse of psoriasis during the follow-up period. A total of 84% patients treated achieved a 100% improvement of the PASI score at 24 weeks and efficacy continued to improve with longer treatment, maintaining same result also during follow-up observation. Moreover, no adverse reactions and no probiotic-related infections were observed.

In conclusion, according to the current guidelines for adults and children affected by mild psoriasis, topical corticoids and vitamin D derivatives can be used alone or in combination. Depending on the form of psoriasis, one or the other treatment can be prescribed. If patients cannot be cured with these treatments due to side effects or comorbidities, the use other possible treatment is allowed. *S. salivarius* is an oral probiotic product, which recently becomes available in Italy for pediatric and adult use that antagonize the growth of *Streptococcus pyogenes* [3, 4]. Based on the results of this longitudinal study, it appears that oral preparations containing *S. salivarius* may provide a beneficial option for the prevention and cure of psoriasis. Their use may be particularly effective in patients who would otherwise be forced to undergo frequent cycles of anti-psoriatic therapy. The literature on probiotic for mild psoriasis is scarce, but we think that they have promising capacity for the management of this disease.

## Data Availability

Data will be available in anonimized form under direct request to the corresponding author.
